# Chelator-Free Labeling of Layered Double Hydroxide Nanoparticles for *in Vivo* PET Imaging

**DOI:** 10.1038/srep16930

**Published:** 2015-11-20

**Authors:** Sixiang Shi, Brianne C. Fliss, Zi Gu, Yian Zhu, Hao Hong, Hector F. Valdovinos, Reinier Hernandez, Shreya Goel, Haiming Luo, Feng Chen, Todd E. Barnhart, Robert J. Nickles, Zhi Ping Xu, Weibo Cai

**Affiliations:** 1 Materials Science Program, University of Wisconsin–Madison, WI, USA; 2 Department of Radiology, University of Wisconsin–Madison, WI, USA; 3 Australian Institute for Bioengineering and Nanotechnology, The University of Queensland, Brisbane, QLD, Australia; 4 Department of Medical Physics, University of Wisconsin–Madison, WI, USA; 5 University of Wisconsin Carbone Cancer Center, Madison, WI, USA

## Abstract

Layered double hydroxide (LDH) nanomaterial has emerged as a novel delivery agent for biomedical applications due to its unique structure and properties. However, *in vivo* positron emission tomography (PET) imaging with LDH nanoparticles has not been achieved. The aim of this study is to explore chelator-free labeling of LDH nanoparticles with radioisotopes for *in vivo* PET imaging. Bivalent cation ^64^Cu^2+^ and trivalent cation ^44^Sc^3+^ were found to readily label LDH nanoparticles with excellent labeling efficiency and stability, whereas tetravalent cation ^89^Zr^4+^ could not label LDH since it does not fit into the LDH crystal structure. PET imaging shows that prominent tumor uptake was achieved in 4T1 breast cancer with ^64^Cu-LDH-BSA via passive targeting alone (7.7 ± 0.1%ID/g at 16 h post-injection; n = 3). These results support that LDH is a versatile platform that can be labeled with various bivalent and trivalent radiometals without comprising the native properties, highly desirable for PET image-guided drug delivery.

Layered double hydroxide (LDH) nanomaterial has emerged as a novel delivery agent and attracted tremendous interest in the past decades. As a natural mineral and readily synthesized material, LDH has a hydrotalcite-like structure, consisting of positively charged brucite-like cationic layers, negatively charged interlayer anions, and hydrogen bonded water molecules, with a general composition formula of [M^2+^_1−x_M^3+^_x_(OH)^2^]^x+^(A^n−^)_x/n_·mH_2_O, where M^2+^, M^3+^ and A^n−^ represent divalent and trivalent metal cation, and anion, respectively^1^,^2^. Due to its unique structure and properties, such as rich surface functionality, excellent biocompatibility, wide availability and controllable ion-exchange, LDH exhibits great potential as a delivery agent for biomedical applications^3^. In regard to diagnostic imaging applications, numerous fluorescent dyes and inorganic nanoparticles have been tethered onto LDH for optical imaging, x-ray computed tomography (CT) and magnetic resonance imaging (MRI)^4^,^5^,^6^,^7^,^8^,^9^,^10^. However, positron emission tomography (PET), which offers great sensitivity, superb tissue penetration, accurate quantification capability and excellent translational potential^11^,^12^,^13^, has not been explored with LDH-based nanoparticles.

To perform PET imaging, radiometals (e.g. ^64^Cu, ^44^Sc and ^89^Zr) constitute a primary category of radioisotopes. The coordination of certain chelators is usually required for the stable labeling of radionuclides^14^,^15^. However, due to the uniqueness of each radionuclide, particular coordination chemistry is necessary, and hence selecting the best chelator and optimal labeling condition becomes an inevitable challenge^15^. On the contrary, chelator-free labeling, which eliminates the need of coordination with chelators, has been considered as a promising labeling approach to nanoparticle-based PET imaging^16^. LDH nanoparticles allow the synchronous incorporation of multiple bivalent and trivalent cations (M^2+^ and M^3+^) into the brucite-like cationic layers^17^. Therefore, we hypothesized that different radionuclides could be incorporated into LDH nanoparticles through a chelator-free manner, providing a novel nanoplatform for versatile radiolabeling.

Recently, we developed a reliable method to produce Mg_2_Al-LDH nanoparticles, which have a well controlled size range (50–300 nm) and can be homogeneously dispersed in the aqueous suspension^18^,^19^. Because LDH nanoparticles are highly positively charged (zeta potential: 30–50 mV)^19^, surface modification can be easily accomplished with negatively charged protein (e.g. bovine serum albumin [BSA]) to prevent possible aggregation in physiological buffer or *in vivo* environment, as recently reported^20^. Therefore, in this study, we propose chelator-free labeling of BSA-modified Mg_2_Al-LDH nanoparticles by simply mixing with a variety of PET isotopes (e.g. ^64^Cu, ^44^Sc and ^89^Zr), followed by evaluation of the labeling yield and stability *in vitro*. *In vivo* PET imaging was subsequently carried out for the first time to investigate the *in vivo* biodistribution of radiolabeled LDH nanoparticles. Rapid and persistent tumor uptake via passive targeting was observed in subcutaneous 4T1 murine breast cancer models.

## Results

### Physicochemical features of LDH nanoparticles

**Figure 1:**
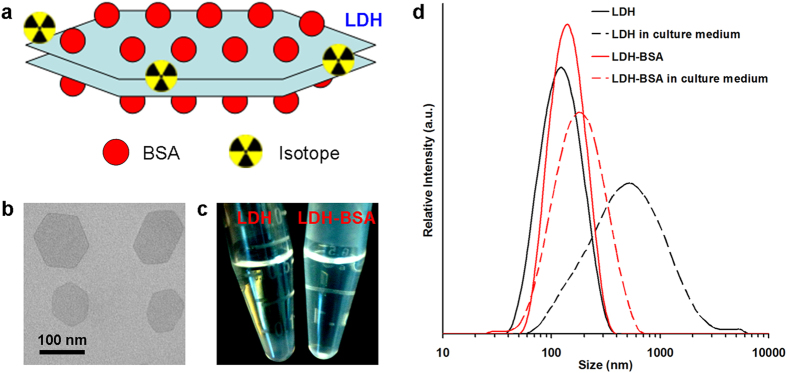
Schematic illustration and characterization of LDH nanoparticles. (**a**) A schematic structure of ^64^Cu-LDH-BSA. **(b)** TEM image of LDH nanoparticles. Scale bar, 100 nm. **(c)** LDH aggregated but LDH-BSA remained stable after incubating LDH and LDH-BSA (4.7 mg/mL) in PBS for 7 days. **(d)** The size distribution of LDH and LDH-BSA in both water and culture media measured by DLS. The size of LDH nanoparticles increased significantly in culture media, whereas the size of LDH-BSA is similar in both water and culture media.

Mg_2_Al-LDH nanoparticles were synthesized via quick precipitation and subsequent hydrothermal treatment as reported previously^1^,^18^,^19^. As shown in the transmission electron microscopy (TEM) image, LDH nanoparticles are 2-dimensional nanomaterials in layered-sheet shape with size in the range of 100-150 nm (Fig. 1b). Dynamic light scattering (DLS) measurement showed that LDH nanoparticles have an average size of 110 nm (polydispersity index [PDI]: 0.18) in water and 380 nm (PDI: 0.44) in culture media (Fig. 1d). The obviously larger size and PDI of LDH in culture media indicate that the unmodified nanoparticles are not very colloidally stable in physiological environment, which restricts the application of LDH in radiolabeling and *in vivo* PET imaging.

To improve the stability of LDH *in vitro* and *in vivo*, negatively charged BSA was used to coat the positively charged LDH surface via electrostatic interactions^20^. After the coating, as-prepared LDH-BSA nanoparticles remained stable in phosphate buffered saline (PBS) for 7 days, whereas LDH without BSA coating precipitated in PBS when incubated for the same duration (Fig. 1c). DLS measurements showed that the average size of LDH-BSA slightly increased to 130 (PDI: 0.21) in water and 150 (PDI: 0.24) in culture media due to the integration of BSA (Fig. 1d), which indicates that LDH-BSA remained stable in both water and PBS, consistent with the observations reported^20^. The final schematic structure of LDH-nanoparticles (^64^Cu-LDH-BSA) is illustrated in Fig. 1a.

### Chelator-free labeling

Different isotopes (^64^Cu, ^44^Sc and ^89^Zr) were directly mixed with LDH-BSA in corresponding buffers (^64^Cu: 0.1 M sodium acetate, pH 5.5; ^44^Sc: 0.5 M sodium acetate, pH 4.5; ^89^Zr: 0.5 M HEPES [4-(2-hydroxyethyl)-1-piperazineethanesulfonic acid], pH 7.0) for chelator-free labeling. LDH nanoparticles without BSA coating and only BSA were also labeled to validate that LDH but not BSA plays a more important role in the labeling process. Thin-layered chromatography (TLC) was used to measure the labeling yield with different isotopes using 50 mM EDTA as the mobile phase, which can remove the unbound and unstably adsorbed isotopes, ensuring that the resulting labeling yield truly represents the capacity of LDH or BSA for chelator-free labeling.

**Figure 2:**
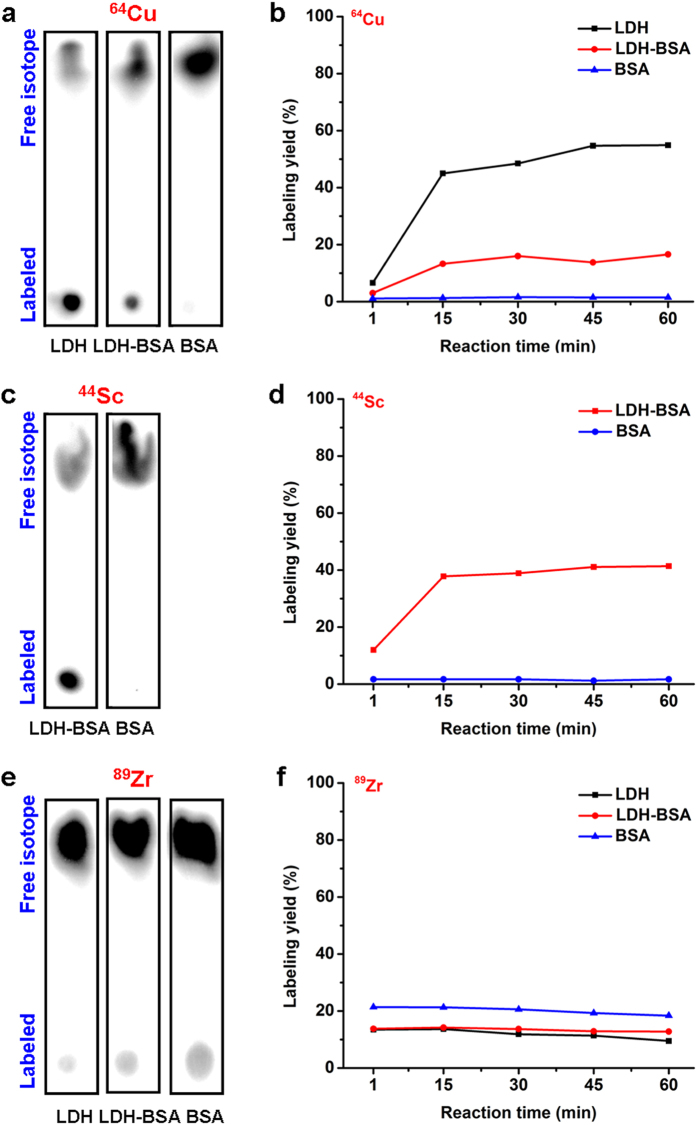
Chelator-free labeling of LDH nanoparticles. **(a,c,e)** Autoradiographic images of TLC plates of LDH, LDH-BSA and BSA after chelator-free labeling with ^64^Cu, ^44^Sc and ^89^Zr for 60 min. **(b**,**d,f**) The labeling yield of LDH, LDH-BSA and BSA after chelator-free labeling with ^64^Cu, ^44^Sc and ^89^Zr at different reaction times calculated from autoradiography images of TLC plates.

^64^Cu^2+^ and ^44^Sc^3+^ were found to successfully labeled LDH and LDH-BSA but not BSA, since LDH allows incorporation of multiple bivalent and trivalent cations^17^. On the contrary, ^89^Zr^4+^ could not label LDH and LDH-BSA, possibly because it does not fit into the LDH crystal structure (Fig. 2; supplementary Fig. 1–3). In detail, after 60 min incubation, 59.0% ^64^Cu^2+^ was incorporated into LDH, while 16.6% was incorporated into LDH-BSA (Fig. 2a,b). The decrease in the labeling capacity of LDH-BSA is possibly attributed to the presence BSA, which prevents the interaction between ^64^Cu^2+^ and LDH. In addition, the stable labeling of ^64^Cu^2+^ on BSA was minimal (~1.5%), indicating that chelator-free labeling of ^64^Cu^2+^ mainly takes place on LDH rather than BSA. Similar results were also obtained with ^44^Sc^3+^. After 60 min incubation, 41.4% ^44^Sc^3+^ was incorporated in LDH-BSA, much higher than that reacting with BSA only (1.7%; Fig. 2c,d). The evaluation of the labeling yield of LDH only with ^44^Sc^3+^ was not possible, due to the aggregation of LDH nanoparticles in high ion-strength buffer (0.5 M sodium acetate) without adequate BSA protection. In comparison, after 60 min of incubation with ^89^Zr^4+^, the labeling yields were similar for all 3 samples (LDH: 9.5%; LDH-BSA: 12.5%; BSA 18.4%; Fig. 2e,f), indicating that ^89^Zr^4+^ cannot efficiently label onto LDH via chelator-free mechanism. Interestingly, ^89^Zr^4+^ could attach onto BSA, which is possibly due to the high affinity of ^89^Zr^4+^ to anionic oxygen donors in BSA^21^,^22^. Of note, chelator-free labeling was very quick, as the labeling yield peaked at 15 min and remained stable at the later time points for all three isotopes (Fig. 2b,d,f). It is of concern that different specific activities of ^64^Cu^2+^, ^44^Sc^3+^ and ^89^Zr^4+^ could be responsible for different radiolabeling outcomes. However, considering that chelator-free labeling takes place on the surface lattice of LDH nanoparticles which in turn provides excessively abundant labeling sites, the different specific activities are not expected to have significant impact on the radiolabeling.

**Figure 3:**
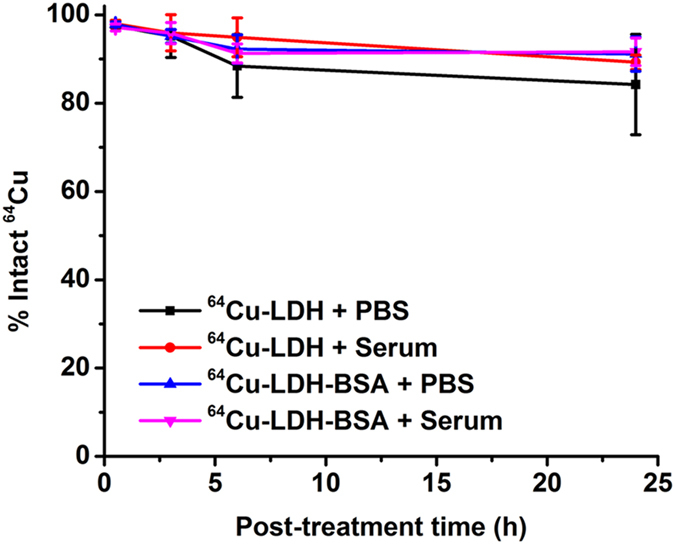
Labeling stability of LDH nanoparticles. Labeling stability was observed with ^64^Cu-LDH and ^64^Cu-LDH-BSA in both PBS and complete mouse serum during 24 h incubation (n = 3).

Labeling stability is another common concern for chelator-free labeling. Besides the usage of EDTA solution during TLC analysis to remove unstably adsorbed ions, the stability of ^64^Cu-LDH and ^64^Cu-LDH-BSA was also tested in PBS and complete mouse serum before administration in living animals. After incubation in PBS for 24 h, 84.2 ± 11.4% and 89.3 ± 1.6% ^64^Cu^2+^ were still stable on LDH and LDH-BSA, respectively (Fig. 3). Similarly, after incubation with complete mouse serum for 24 h, 91.1 ± 3.9% and 91.6 ± 3.1% ^64^Cu^2+^ were stable on LDH and LDH-BSA, respectively (Fig. 3). These data demonstrate successful and stable chelator-free labeling of LDH and LDH-BSA nanoparticles.

### *In vivo* PET and biodistribution studies

**Figure 4:**
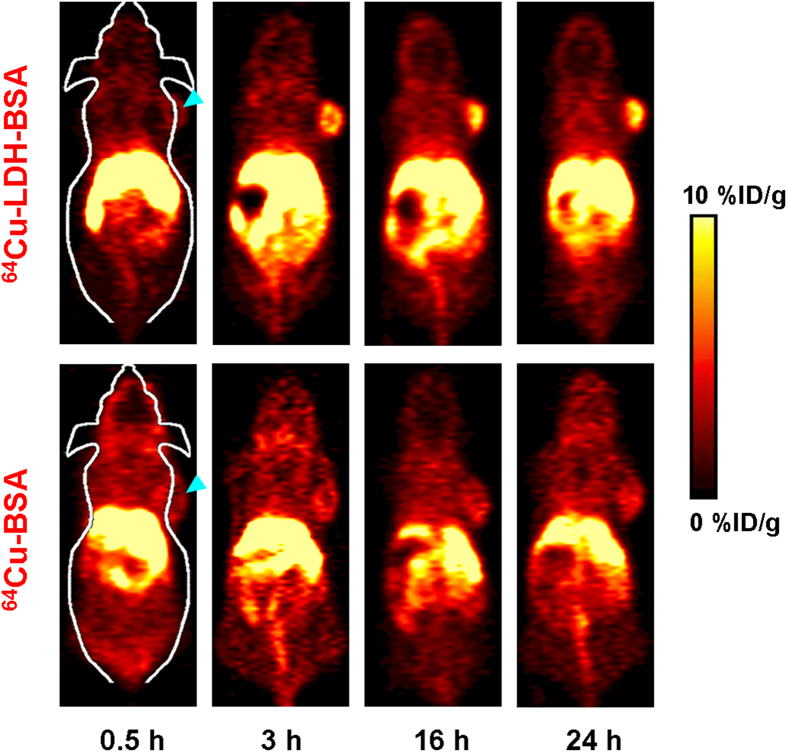
*In vivo* PET imaging. Serial coronal PET images at different time points post-injection of ^64^Cu-LDH-BSA and ^64^Cu-BSA were acquired in 4T1 tumor-bearing mice. Strong signal in tumor was observed in the mice injected with ^64^Cu-LDH-BSA. Three mice were scanned per group (n = 3).

**Figure 5:**
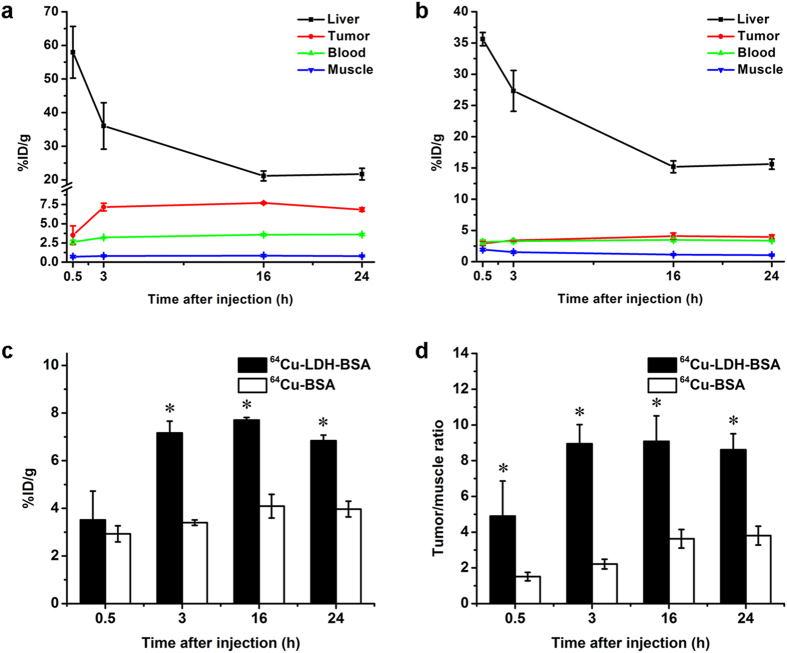
Quantitative analysis of the PET data. **(a)** Time activity curves of the liver, 4T1 tumor, blood, and muscle upon intravenous injection of ^64^Cu-LDH-BSA. **(b)** Time activity curves of the liver, 4T1 tumor, blood, and muscle upon intravenous injection of ^64^Cu-BSA. **(c)** Comparison of tumor uptake at different time points post injection of ^64^Cu-LDH-BSA and ^64^Cu-BSA. The differences of the tumor uptake were statistically significant (P < 0.05) at all time points except 0.5 h. (**d)** Comparison of tumor/muscle ratio at different time points post injection of ^64^Cu-LDH-BSA and ^64^Cu-BSA. All data were back-decayed to the injection time. The differences of tumor/muscle ratio were statistically significant (P < 0.05) at all time points. All data represent 3 mice per group (n = 3).

After purification with PD-10 size exclusion column chromatography, ^64^Cu-LDH-BSA was intravenously injected into 4T1 tumor-bearing mice to examine their *in vivo* distribution profile. PET imaging was performed at different time points (0.5 h, 3 h, 16 h and 24 h) post-injection (p.i.) using a microPET/microCT Inveon rodent model scanner. The coronal PET images are shown in Fig. 4 and quantitative region-of-interest (ROI) analysis is shown in Fig. 5. A prompt and persistent tumor uptake (3.5 ± 1.2, 7.2 ± 0.5, 7.7 ± 0.1 and 6.8 ± 0.2%ID/g, at 0.5 h, 3 h, 16 h and 24 h p.i., respectively; n = 3; Fig. 5a,c; supplementary Table 1) was achieved via passive targeting based on enhanced permeability and retention (EPR) effect. In contrast, the probe retentions in blood and muscle were much lower at all time points (blood: 2.6 ± 0.2, 3.2 ± 0.1, 3.6 ± 0.1 and 3.6 ± 0.1%ID/g, and muscle: 0.7 ± 0.1, 0.8 ± 0.1, 0.8 ± 0.1 and 0.8 ± 0.1%ID/g, at 0.5 h, 3 h, 16 h and 24 h p.i., respectively; n = 3; Fig. 5a; supplementary Table 1). Tumor/muscle ratios of 4.9 ± 2.0, 8.9 ± 1.1, 9.1 ± 1.4 and 8.6 ± 0.9 were achieved at 0.5 h, 3 h, 16 h and 24 h p.i., respectively (n = 3; Fig. 5d; supplementary Table 3), indicative of an excellent tumor contrast. Furthermore, the signal in liver peaked at early time point and decreased with time (58.0 ± 7.7, 36.0 ± 6.9, 21.2 ± 1.5 and 21.7 ± 1.7%ID/g, at 0.5 h, 3 h, 16 h and 24 h p.i., respectively; n = 3; Fig. 5a; supplementary Table 1), indicating hepatic clearance (bile to feces) of LDH nanoparticles. Taken together, with a prominent tumor uptake and excellent image contrast, ^64^Cu-LDH-BSA has proven to be a promising nanoplatform for *in vivo* tumor imaging via passive targeting upon chelator-free labeling. With further surface engineering, it could also be applied to active tumor targeting and therapy.

One concern with *in vivo* PET imaging with ^64^Cu-LDH-BSA is the possible detachment of BSA from LDH nanoparticles in blood stream. To investigate the influence of possibly detached of BSA on nanoparticle pharmacokinetics and tumor uptake, PET imaging was also performed with ^64^Cu-BSA, although the labeling yield of ^64^Cu-BSA is much lower than that of ^64^Cu-LDH-BSA. After intravenous injection of ^64^Cu-BSA into 4T1 tumor-bearing mice, a significantly lower tumor uptake was observed (2.9 ± 0.3, 3.4 ± 0.1, 4.1 ± 0.5 and 4.0 ± 0.3%ID/g, at 0.5 h, 3 h, 16 h and 24 h p.i., respectively; n = 3; Fig. 4 and Fig. 5b,c; supplementary Table 2). Moreover, the background signal in muscle was higher (1.9 ± 0.2, 1.5 ± 0.2, 1.1 ± 0.1 and 1.0 ± 0.1%ID/g, at 0.5 h, 3 h, 16 h and 24 h p.i., respectively; n = 3; Fig. 5b; supplementary Table 2), possibly due to the smaller size and slower clearance of ^64^Cu-BSA compared with ^64^Cu-LDH-BSA, which led to a poorer imaging contrast (tumor/muscle ratio:1.5 ± 0.2, 2.2 ± 0.3, 3.6 ± 0.5 and 3.8 ± 0.5, at 0.5 h, 3 h, 16 h and 24 h p.i., respectively; n = 3; Fig. 5d; supplementary Table 3). Combining these data and the fact that BSA has a significantly lower labeling yield than that of LDH, we could draw a preliminary conclusion that the prominent tumor uptake of ^64^Cu-LDH-BSA was primarily due to LDH, and any possible detachment of BSA *in vivo* will not significantly influence the imaging results.

**Figure 6:**
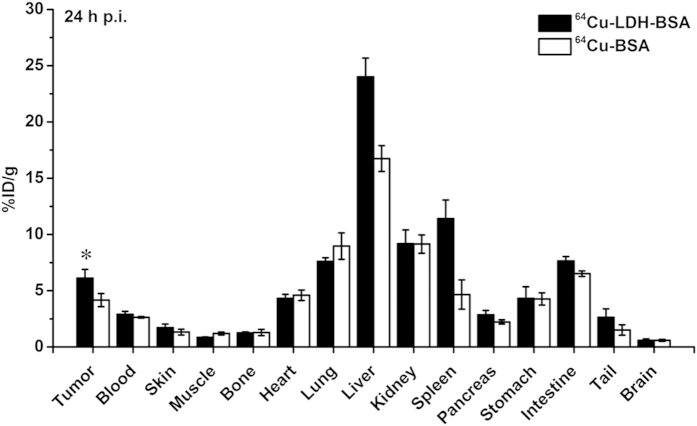
Biodistribution of ^64^Cu-LDH-BSA and ^64^Cu-BSA in 4T1 tumor-bearing mice. The tumor uptake of ^64^Cu-LDH-BSA was significantly higher than that of ^64^Cu-BSA at 24 h post injection (P < 0.05; n = 3).

After the last scan at 24 h p.i., 4T1 tumor-bearing mice injected with ^64^Cu-LDH-BSA and ^64^Cu-BSA were sacrificed for biodistribution studies. The quantitative data of the uptake in tumor and other tissues in biodistribution studies matched well with the results of ROI analysis, which confirmed the accuracy of PET imaging and ROI analysis (Fig. 6).

## Discussion

Compared with conventional chelator-based labeling, chelator-free labeling possesses several unique advantages. First, chelator-free labeling of nanoparticles is a versatile approach, which may be suitable for various radionuclides. Therefore, a single design or strategy can potentially be employed in the different clinical settings, as exemplified by our current study. Secondly, it is known that incorporation of chelators could sometimes alter the *in vivo* biodistribution and pharmacokinetics of nanoparticles, which may not accurately reflect the pharmacological behavior of unlabeled nanoparticles^23^,^24^,^25^. Chelator-free labeling which avoids the use of a chelating agent, therefore, can maintain the native pharmacokinetic profile of the nanoparticles. Thirdly, chelator-free labeling overcomes the limitation of specific activities of radionuclides. Unlike traditional labeling procedures which depend on a limited number of chelators conjugated to the nanoparticles, chelator-free labeling takes place directly on the surface (or within the lattice sites) of the nanoparticles via ion-exchange or other mechanisms. Therefore, abundant labeling sites are available resulting in high labeling yields, even though the specific activity is relatively low. Fourthly, chelator-free labeling does not require functional groups on the surface of nanoparticles for conjugation of chelators. Therefore, all the surface functional groups on the nanoparticles can be employed for further functionalization with fluorescent agents, therapeutic agents or targeting ligands to generate multifunctional nanoplatforms. And lastly, the mechanism for chelator-free labeling could be very simple and effective, allowing the labeling of isotopes such as ^72^As and ^69^Ge^15^,^16^,^26^, which to date is not easily achievable with a traditional chelator-based approach. Both ^72^As and ^69^Ge proved very challenging to label via conventional techniques, with few reported successes in the literature^15^,^26^.

However, chelator-free labeling also has certain drawbacks. Firstly, the surface modification of nanoparticles can significantly affect the labeling yield, since the surface of nanoparticles directly interacts with the isotopes during labeling. For example, BSA coating decreased the labeling yield of LDH-BSA with ^64^Cu in this study. In addition, the labeling stability through chelator-free manner varies from case to case, differing for each isotope-nanoparticle combination. Since it is still a relatively underexplored area of research, very few examples can be referred to. In our previous study on chelator-free labeling of *As, obvious bladder uptake was observed in PET images with *As-SPION, which indicates that the labeling was not stable *in vivo*^15^. However, in this study, no bladder uptake was observed *in vivo*, suggesting a great potential of LDH nanoparticles for chelator-free labeling with excellent stability.

In this proof-of-principle study, ion-exchange is believed to play an important role in chelator-free labeling of LDH nanoparticles^27^. Through interactions between LDH nanoparticles and radiometals, the radioisotopes (guest species) tend to exchange with surface LDH atoms (host species) or compensate for the defect of LDH^27^,^28^. This allows the incorporation of various divalent and trivalent metal cations but not tetravalent metal cations, since only divalent and trivalent cations can fit into the LDH crystal lattice ([M^2+^_1−x_M^3+^_x_(OH)^2^]^x+^(A^n−^)_x/n_·mH_2_O). However, further studies are needed to better understand the mechanism of chelator-free labeling in LDH nanoparticles. The valence of radioisotopes might not be the only decisive factor for this chelator-free labeling. Other factors, such as labeling buffer, temperature, pH value, concentration, and particle size, might also influence the labeling results^28^,^29^.

For nanoparticle-related imaging, toxicity of the nanoparticles is always one of the major concerns. Of note, LDH is considered one of the inorganic nanoparticles with low toxicity^30^. The toxicity of LDH is usually dose and time dependent, and the shape, size and surface charge also play a role in nanoparticle toxicity *in vitro* and *in vivo*^27^. In addition, aggregation or agglomeration of LDH nanoparticles upon suspension in physiological fluids is another reason for possible toxicity^3^,^31^. In this respect, BSA was coated onto LDH via electrostatic interactions, which is a straightforward and efficient process. The stability of LDH-BSA was significantly increased to prevent the possible aggregation *in vivo*. Besides BSA, other molecules, such as polyethylene glycol (PEG) or Tween-80, have also been used to modify LDH nanoparticles, which exhibited reduced toxicity in comparison with uncoated ones as previously reported^32^,^33^. Although LDH-nanoparticles have been proven to be highly biocompatible *in vitro* and many approaches have been investigated to modify the LDH surface for reduced potential toxicity, a long-term *in vivo* toxicity test is still of significance and warranted, considering that some elements (e.g. Al) can possibly lead to side effects if taken above a certain quantity or in the presence of certain physiological environments^3^,^34^,^35^,^36^.

The liver uptake is also a major concern for nanoparticle-based PET imaging. After intravenous injection into tumor-bearing mice, the nanoparticles are rapidly sequestered from the blood and accumulated in reticuloendothelial system (RES) organs (e.g. liver and spleen), and then gradually excreted by hepatic pathway (bile to feces)^37^. Depending on the material, size and surface charge of the nanoparticles, the excretion process may take hours to weeks. In this study, hepatic clearance of ^64^Cu-LDH-BSA was evidenced by the decreasing liver uptake over time and radioactivity detected in the feces of mice. Due to relatively high signal in liver, renally non-clearable nanoparticles are not suitable for imaging liver tumors and liver metastases, although nanoparticles possess other advantages such as multimodality and theranostic potential. To reduce the liver uptake, great efforts have been made to improve surface modification of nanoparticle-based imaging agents^38^, which will be also critical for broadening the *in vivo* applications of LDH nanoparticles.

In conclusion, we report the chelator-free labeling and *in vivo* PET imaging with LDH nanoparticles. Upon surface modification with BSA, bivalent cation ^64^Cu^2+^ and trivalent ^44^Sc^3+^ cation successfully labeled LDH nanoparticles with excellent labeling efficiency and stability. Prompt and persistent tumor uptake was also observed with ^64^Cu-LDH-BSA via passive targeting. Without compromising the native properties (e.g. drug loading), LDH can potentially serve as a versatile platform for PET imaging and drug delivery.

## Methods

### Materials

Chelex 100 resin (50–100 mesh) was acquired from Sigma-Aldrich (St. Louis, MO). ^64^Cu was produced in a GE PETrace cyclotron using the ^64^Ni(p,n)^64^Cu reaction, which has a specific activity of ~185 MBq/nmol at the end of bombardment. ^44^Sc was produced in a GE PETrace cyclotron using the ^44^Ca(p,n)^44^Sc reaction, with a specific activity of ~8.2 MBq/nmol at the end of bombardment. ^89^Zr was produced in a GE PETrace cyclotron using the ^89^Y(p,n)^89^Zr reaction, with a specific activity of ~20.0 MBq/nmol at the end of bombardment. Complete mouse serum was acquired from Jackson Immuno Research Laboratories (West Grove, PA). Water and all buffers were of Millipore grade and pretreated with Chelex 100 resin to ensure that the aqueous solution was free of heavy metals. PD-10 desalting columns were acquired from GE Healthcare (Piscataway, NJ). All other reaction buffers and chemicals were from Thermo Fisher Scientific.

### Cell lines and animal models

4T1 murine breast cancer cells were obtained from American Type Culture Collection (ATCC, Manassas, VA) and cultured according to the supplier’s instructions. When they reached ~80% confluence, the cells were collected for tumor implantation^39^. Four- to five-week-old female BALB/c mice (Harlan, Indianapolis, IN) were each injected with 2 × 10^6^ 4T1 cells in the flank to generate the 4T1 breast cancer model. The mice were used for *in vivo* experiments when the tumor diameter reached 6–8 mm. All animal studies were conducted under a protocol approved by the University of Wisconsin Institutional Animal Care and Use Committee.

### Synthesis of LDH

Mg_2_Al-based LDH nanoparticles were synthesized using a quick precipitation and subsequent hydrothermal treatment as reported previously^1^,^18^,^19^. In brief, 3.0 mmol of MgCl_2_·6H_2_O and 1.0 mmol of AlCl_3_·6H_2_O were dissolved in 10 mL deionized water, and then rapidly added to a basic solution (40 mL) containing 6.0 mmol of NaOH and 0.6 mmol of Na_2_CO_3_ within 5 s. As-prepared solution was then stirred in N_2_ stream at room temperature for 10 min. Subsequently, the precipitate was collected and washed twice with deionized water via centrifugation, and re-dispersed in 15 mL of deionized water and placed in a 20 mL autoclave with Teflon linen. Lastly, hydrothermal treatment was carried out at 100 °C in an oven for 16 h to generate the final suspension of Mg_2_Al-LDH nanoparticles with the mass concentration of 14.0 mg/mL.

### BSA coating of LDH

0.2 ml of Mg_2_Al–CO_3_ LDH nanoparticles (14.0 mg/mL) were slowly and dropwise added to 0.4 ml BSA solution (50 mg/mL) under vigorous stirring. After stirring for 2 h at room temperature, the solution was centrifuged (4,000 rpm for 10 min) and washed with PBS for multiple times. As-prepared LDH-BSA suspension contained 4.7 mg/mL of LDH and 33.3 mg/mL of BSA. Both LDH and LDH-BSA nanoparticles were characterized with TEM and DLS to measure their morphology and size.

### Radiolabeling and labeling stability

^64^Cu, ^44^Sc, and ^89^Zr were produced in an on-site cyclotron (GE PETrace). ^64^CuCl_2_ (74 MBq), ^44^ScCl_3_ (74 MBq), and ^89^Zr-oxalate (74 MBq) were diluted in 300 μL of 0.1 M sodium acetate buffer (pH 5.5), 0.5 M sodium acetate buffer (pH 4.5) and 0.5 M HEPES (4-(2-hydroxyethyl)-1-piperazineethanesulfonic acid) buffer (pH 7.0), respectively^39^,^40^,^41^. The diluted solutions were then mixed with 50 μL of LDH (4.7 mg/mL), LDH-BSA (4.7 mg/mL based on LDH concentration) or only BSA (33.3 mg/ml, the same concentration as that in LDH-BSA) for chelator-free labeling. The labeling reaction was carried out at 37 °C for 60 min under constant shaking. The labeling yield was measured by TLC using 50 mM ethylenediaminetetraacetic acid (EDTA) as the mobile phase to eliminate unstable adsorption of isotopes. The labeling yields at different reaction time were calculated from autoradiography images of TLC plates. The radioactive fraction was purified with PD-10 size exclusion column chromatography using PBS as the mobile phase.

To test the labeling stability, ^64^Cu-LDH and ^64^Cu-LDH-BSA were incubated in both PBS and complete mouse serum at 3 °C for up to 24 h under constant shaking. Portions of the mixture were sampled at different time points and filtered through 100 kDa MWCO filters. The radioactivity that remained on the filter was measured after discarding the filtrate, and retained (i.e., intact) ^64^Cu-LDH or ^64^Cu-LDH-BSA was calculated using the equation (radioactivity on filter/total sampling radioactivity ×100%).

### *In vivo* PET and biodistribution studies

Serial PET scans of ^64^Cu-LDH-BSA were performed in 4T1 tumor-bearing mice (n = 3) using a microPET/microCT Inveon rodent model scanner (Siemens Medical Solutions USA, Inc.), at different time points (0.5 h, 3 h, 16 h , and 24 h) post-injection (p.i.) of ^64^Cu-LDH-BSA via tail vein. Data acquisition, image reconstruction, and region-of-interest (ROI) analysis of the PET data were performed as previously described^13^,^42^,^43^. Quantitative data of ROI analysis on tumor and other organs were presented as percentage injected dose per gram of tissue (%ID/g). To validate the labeling and imaging of ^64^Cu-LDH-BSA, ^64^Cu-BSA was also scanned in 4T1 tumor-bearing mice (n = 3) as a control group. After the last scan at 24 h p.i., mice were sacrificed under anesthesia for biodistribution studies. Tumor, blood and major organs/tissues were collected and weighted. The radioactivity in the tissue was measured using a γ counter (PerkinElmer) and presented as %ID/g (mean ± SD).

## Additional Information

**How to cite this article**: Shi, S. *et al.* Chelator-Free Labeling of Layered Double Hydroxide Nanoparticles for *in Vivo* PET Imaging. *Sci. Rep.* **5**, 16930; doi: 10.1038/srep16930 (2015).

## Supplementary Material

Supplementary Information
