# Supplementary material for: Chelator-Free Labeling of Layered Double Hydroxide Nanoparticles for in Vivo PET Imaging
Source: Sci Rep. 2015 Nov 20;5:16930. doi: 10.1038/srep16930 (PMC4653656; doi:10.1038/srep16930)
Supplement: Supplementary Information [file srep16930-s1.doc]

**Chelator-Free Labeling of Layered Double Hydroxide Nanoparticles for in Vivo PET Imaging**

Sixiang Shi^1^, Brianne Fliss^2^, Zi Gu^3^, Yian Zhu^3^, Hao Hong^2^, Hector F. Valdovinos^4^, Reinier Hernandez^4^, Shreya Goel^1^, Haiming Luo^2^, Feng Chen^2^, Todd E. Barnhart^4^, Robert J. Nickles^4^, Zhi Ping Xu^3^, and Weibo Cai^1,2,4,5^

^1^ Materials Science Program, University of Wisconsin–Madison, WI, USA

^2^ Department of Radiology, University of Wisconsin–Madison, WI, USA

^3^ Australian Institute for Bioengineering and Nanotechnology, The University of Queensland, Brisbane, QLD, Australia

^4^ Department of Medical Physics, University of Wisconsin–Madison, WI, USA

^5^ University of Wisconsin Carbone Cancer Center, Madison, WI, USA

Sixiang Shi and Brianne Fliss contributed equally to this work.

**SUPPLEMENTARY INFORMATION**

**Supplemental Figure 1. Chelator-free labeling of ^64^Cu on LDH nanoparticles.** Autoradiographic images of TLC plates of LDH, LDH-BSA and BSA after 1, 15, 30, 45 and 60 min reaction with ^64^Cu.

**Supplementary Figure 2. Chelator-free labeling of ^44^Sc on LDH nanoparticles.** Autoradiographic images of TLC plates LDH-BSA and BSA after 1, 15, 30, 45 and 60 min reaction with ^44^Sc. Chelator-free labeling of ^44^Sc with LDH without BSA coating was not performed due to aggregation in the labeling buffer.

**Supplementary Figure 3. Chelator-free labeling of ^89^Zr on LDH nanoparticles.** Autoradiographic images of TLC plates of LDH, LDH-BSA and BSA after 1, 15, 30, 45 and 60 min reaction with ^89^Zr.

Supplementary Table 1. Quantitative PET analysis of ^64^Cu-LDH-BSA (n = 3).

|  | 0.5 h  (%ID/g) | 3 h  (%ID/g) | 16 h  (%ID/g) | 24 h  (%ID/g) |
| --- | --- | --- | --- | --- |
| Liver | 58.0 ± 7.7 | 36.0 ± 6.9 | 21.2 ± 1.5 | 21.7 ± 1.7 |
| Tumor | 3.5 ± 1.2 | 7.2 ± 0.5 | 7.7 ± 0.1 | 6.8 ± 0.2 |
| Blood | 2.6 ± 0.2 | 3.2 ± 0.1 | 3.6 ± 0.1 | 3.6 ± 0.1 |
| Muscle | 0.7 ± 0.1 | 0.8 ± 0.1 | 0.8 ± 0.1 | 0.8 ± 0.1 |

Supplementary Table 2. Quantitative PET analysis of ^64^Cu-BSA (n = 3).

|  | 0.5 h  (%ID/g) | 3 h  (%ID/g) | 16 h  (%ID/g) | 24 h  (%ID/g) |
| --- | --- | --- | --- | --- |
| Liver | 35.6 ± 1.1 | 27.3 ± 3.3 | 15.2 ± 0.9 | 15.6 ± 0.8 |
| Tumor | 2.9 ± 0.3 | 3.4 ± 0.1 | 4.1 ± 0.5 | 4.0 ± 0.3 |
| Blood | 3.2 ± 0.3 | 3.3 ± 0.3 | 3.5 ± 0.31 | 3.4 ± 0.1 |
| Muscle | 1.9 ± 0.2 | 1.5 ± 0.2 | 1.1 ± 0.1 | 1.0 ± 0.1 |

Supplementary Table 3. Tumor-to-muscle ratios at different post-injection time points. (n = 3).

|  | 0.5 h | 3 h | 16 h | 24 h |
| --- | --- | --- | --- | --- |
| 64Cu-LDH-BSA | 4.9 ± 2.0 | 8.9 ± 1.1 | 9.1 ± 1.4 | 8.6 ± 0.9 |
| 64Cu-BSA | 1.5 ± 0.2 | 2.2 ± 0.3 | 3.6 ± 0.5 | 3.8 ± 0.5 |
